# Idiopathic Pulmonary Hemosiderosis in Adults: A Case Report and Review of the Literature

**DOI:** 10.1155/2012/267857

**Published:** 2012-07-18

**Authors:** Argyris Tzouvelekis, Paschalis Ntolios, Anastasia Oikonomou, Anastasios Koutsopoulos, Efthimios Sivridis, George Zacharis, Kostantinos Kaltsas, Panagiotis Boglou, Dimitrios Mikroulis, Demosthenes Bouros

**Affiliations:** ^1^Department of Pneumonology, University Hospital of Alexandroupolis, Democritus University of Thrace, 68100 Alexandroupolis, Greece; ^2^Centre for Respiratory Research, Division of Medicine, University College London, London WC1E6JE, UK; ^3^Department of Radiology, University Hospital of Alexandroupolis, Democritus University of Thrace, 68100 Alexandroupolis, Greece; ^4^Department of Pathology, University Hospital of Alexandroupolis, Democritus University of Thrace, 68100 Alexandroupolis, Greece; ^5^Department of Cardiac and Thoracic Surgery, University Hospital of Alexandroupolis, Democritus University of Thrace, 68100 Alexandroupolis, Greece

## Abstract

Idiopathic pulmonary hemosiderosis is a very rare condition rarely affecting adults and causing recurrent episodes of diffuse alveolar haemorrhage that may lead to lung fibrosis. Due to lack of pathognomonic findings, IPH diagnosis is established upon exclusion of all other possible causes of DAH in combination with specific pathologic findings revealing bland alveolar haemorrhage with absence of vasculitis and/or accumulation of immune complexes within lung parenchyma. Here we describe a rare case of idiopathic pulmonary hemosiderosis in an otherwise healthy 27-year-old Greek male patient with relapsing episodes of fever accompanied by general fatigue and discomfort. He was at this time point a light smoker and had been hospitalised once in the past for similar symptoms. His iron deficiency anemia coupled with chest high-resolution computed tomography and bronchoalveolar lavage revealed findings compatible with diffuse alveolar hemorrhage. After excluding all other sources of bleeding through extensive gastrointestinal workup and thorough immunologic profile, video-assisted thoracic lung biopsy was performed and the diagnosis of Idiopathic Pulmonary Hemosiderosis was established. Patient was treated with high doses of oral corticosteroids, leading to clinical response. We highlight the need for vigilance by the respiratory physician for the presence of DAH, a challenging, acute condition requiring early recognition along with identification of the underlying syndrome and appropriate treatment to achieve optimal results.

## 1. Introduction

Diffuse alveolar haemorrhage (DAH) is an emergency and often life-threatening clinical setting. It is the result of the destruction of the pulmonary microvasculature and the subsequent blood extravasation into the alveolar space. Multiple clinical syndromes and conditions may be responsible for the loss of the alveolar capillary bed and intrapulmonary bleeding,including both systemic and limited to the lung vasculitis or capillaritis as well as “bland” pulmonary haemorrhage (without vasculitis or capillaritis). Pulmonary haemorrhage can also complicate systemic diseases like connective tissue disease, Wegener's granulomatosis, pulmonary embolism, or even sarcoidosis [1–3].

Idiopathic pulmonary hemosiderosis (IPH) is a rare cause of DAH. The diagnosis of IPH requires elimination of all other causes and lung biopsy confirmation [4]. Although it mostly affects children, there are also reports of IPH in adults. IPH presents with a wide range of clinical manifestations ranging from exertional dyspnoea and fatigue to more typical findings such as haemoptysis attributed to the intrapulmonary bleeding and the sequential iron deficiency anaemia. Chest X-ray and computed tomography are usually nonspecific and bronchoalveolar lavage (BAL) usually sets the primary diagnosis of DAH [4, 5] (Figure 2).

Here we describe a case of IPH with unusual presentation in an adult male patient without haemoptysis or underlying lung disease, known exposures or symptoms, and laboratory tests indicating any alternative diagnosis that could cause alveolar bleeding. The patient was referred to our centre due to recurrent episodes of fever, regressing with corticosteroids and antibiotics treatment but appearing again after treatment discontinuation.

## 2. Case Presentation

A 27-year-old Greek male patient was presented to the outpatient clinic of the Pulmonary Medicine Department of our hospital due to relapsing episodes of fever spikes (up to 38.7°C) accompanied by general fatigue and discomfort. He was initially treated with a course of oral antibiotics (b lactam plus macrolide) with poor clinical response. He was then transferred to our department for further evaluation. He reported a hospitalization 2 years ago due to fever of unknown origin, which subsided after a short course of high doses of corticosteroids. Nonetheless, he failed to recall any further details regarding the etiology of his hospitalization. He was a current light smoker (5 pack-years) and reported no exposure to illicit drugs, environmental, and occupational allergens or toxic fumes, chemicals, and dust. On physical examination, he was febrile with general fatigue and discomfort; however, he reported no dyspnea, tachypnea (respiratory rate 12 breaths/min), or palpitations (heart rate within normal range-70 bpm). He had no hypoxaemia (partial pressure ofoxygen 84 mm Hg) on arterial blood gas analysis. He had no clubbing, skin lesions, cervical lymphadenopathy, or joint swelling. Auscultation of the lungs mild end-inspiratory crackles in both lower lung zones. Cardiovascular, abdominal, and neurological system examinations were unremarkable.

Laboratory tests were performed and divulged microcytic iron deficiency anaemia (Hct: 35.9%, Hgb: 11.9 g/dL, MCV: 69.7, Fe: 19 mg/dL). Ferritin, B12, and folic acid levels were within normal range. The erythrocyte sedimentation rate was 65 mm·h^−1^. The rest of the physical examination and routine laboratory tests, including white blood cell count and differential, red blood cell count, liver and renal function, and serum C-reactive protein, were normal. Gross and microscopic urinary analysis revealed neither hematuria nor renal red blood cell casts, while his 24-hour urine protein levels were within normal range (60 mg/dl). His chest X-ray showed alveolar infiltrates in both lower lobes. Patient was then commenced on intravenous course of broad spectrum of antibiotics (piperacillin/tazobactam plus moxifloxacin) coupled with oseltamivir 75 mgr twice daily, which was discontinued three days after the sputum smear was negative for H1N1.

Laboratory tests for collagen vascular disease and vasculitis, including antinuclear (ANA), antiextractable nuclear antigens (ENA), antineutrophil cytoplasm (ANCA), and antiglomerular basement membrane antibodies, were also negative. The tuberculin skin test was negative. His HIV test was also negative, while his hepatitis B and C antibody titers were within normal range. The patient had a positive Mayer stool test for erythrocytes raising a suspicion for inflammatory bowel disease, but his further gastrointestinal workup (gastroscopy and colonoscopy) excluded any source of bleeding. In particular, gastroduodenal biopsies were performed and findings were inconsistent with both inflammatory bowel disease and malignancy as well as celiac disease. Regarding the latter specific antibodies against transglutaminase (ATA), both IgA and IgG isoforms were also negative. 

Pulmonary function tests (PFTs) were performed and showed normal FVC (91%), FEV_1_ (92%), and FEV_1_/FVC ratio (84.1%) and a strikingly elevated DL_CO_ (120% of the predicted normal value) indicative of alveolar hemorrhage. 

**Figure 1:**
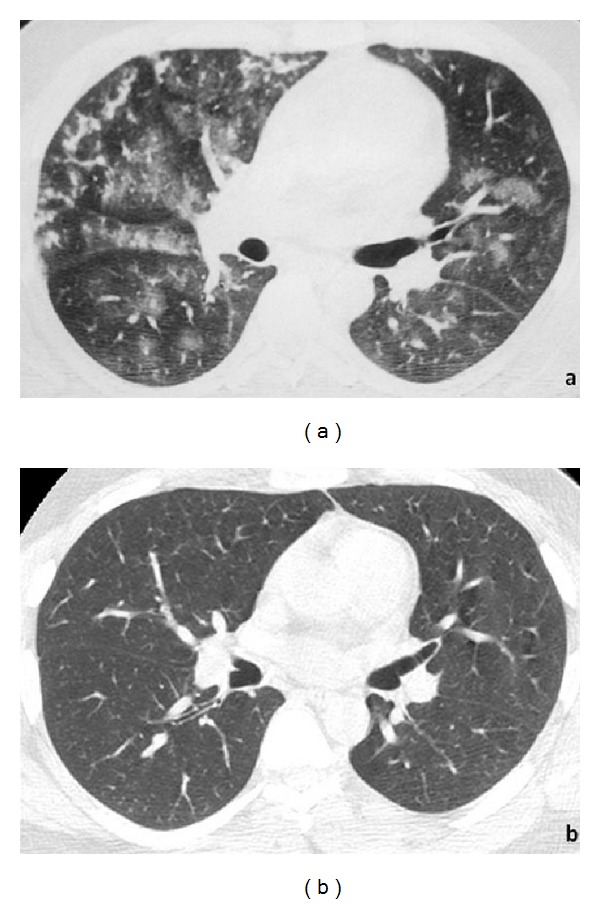
HRCT scan at the level of middle lobe at presentation (a) shows geographic and nodular areas of ground-glass opacity bilaterally as well as branching centrilobular micronodules in the middle lobe consistent with alveolar hemorrhage. HRCT scan following two month corticosteroid treatment at the same level shows complete resolution of the above mentioned findings (b).

**Figure 2:**
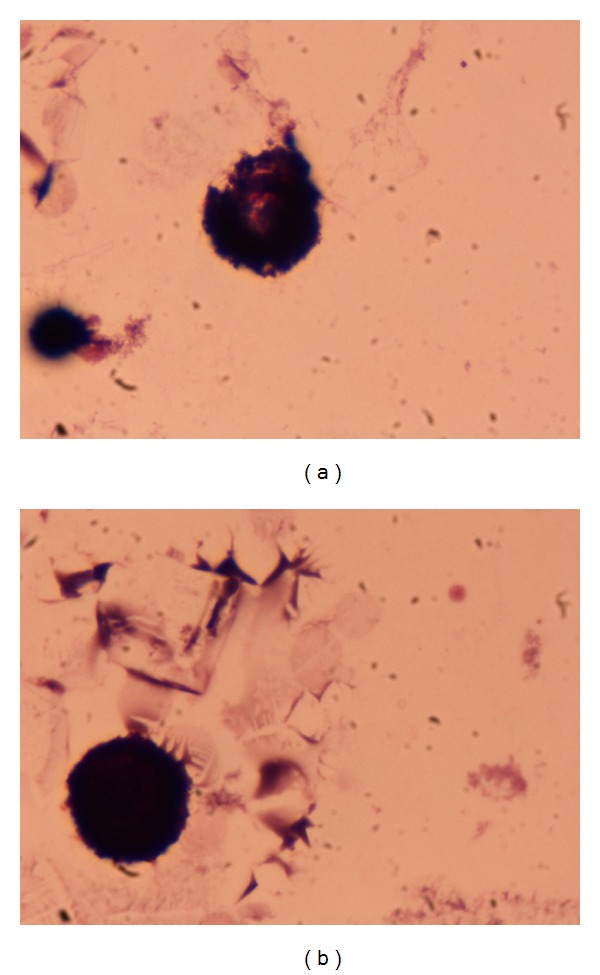
Bronchoalveolar lavage fluid showing numerous hemosiderin-laden macrophages (siderophages) stained positive with Prussian blue ((a) and (b)) as well as clusters of destroyed erythrocytes (b) indicative of alveolar hemorrhage. Cytometric analysis revealed almost 40% of siderophages of the total number of alveolar macrophages.

Patient was then subjected to high-resolution computed tomography (HRCT) showing diffuse bibasilar ground-glass opacities consistent with alveolar hemorrhage (Figure 1). Diagnosis of DAH was confirmed by BAL demonstrating increased numbers of hemosiderin-laden macrophages (>40% of total number of macrophages). BAL fluid specimens were negative for routine bacterial, mycobacterial, fungal, and viral as well as Pneumocystis stains and cultures. Due to the patient's continuously worsening clinical condition (onset of dyspnoea, ESR and CRP increase to 187 mm/h and 15.91 mg/L, constant decrease of haemoglobin levels), a VATS biopsy from the right middle lobe was employed to address the cause of the alveolar haemorrhage. Extensive pathologic evaluation of the lung specimen divulged hemosiderin-laden alveolar macrophages and absence of any specific pathology such as granulomas or evidence of vasculitis/capillaritis. Immunofluorescence microscopy of frozen tissue samples, using a panel of antibodies against complement and immunoglobulins was without notice of immune complexes that would drive a diagnosis towards a specific cause. Taking into consideration the above data, we came up with the diagnosis of IPH.

**Figure 3:**
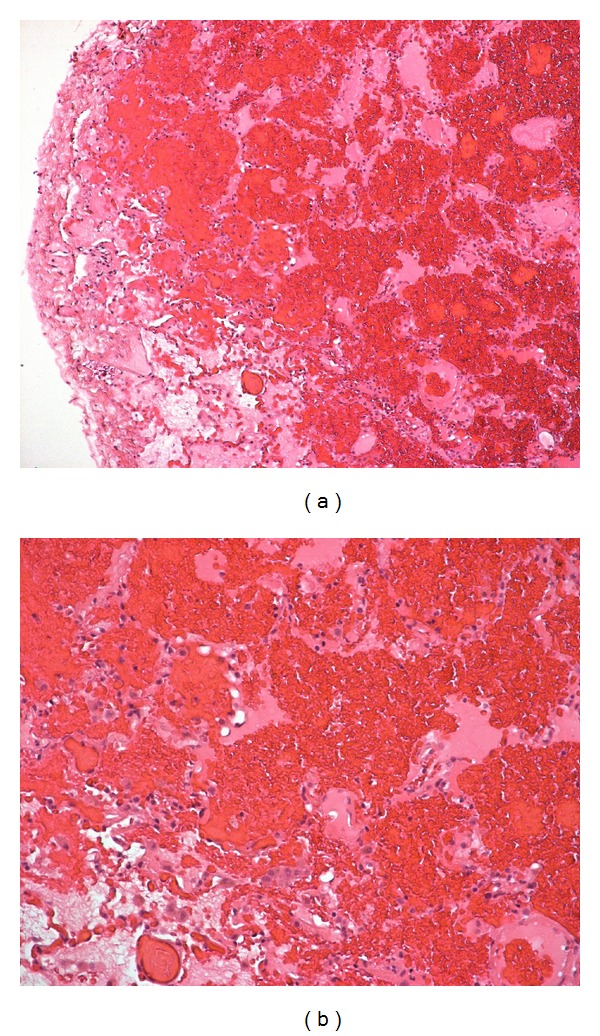
Tissue sections ((a) and (b)) from the lung biopsy showed diffuse intra-alveolar red blood cell. There was, also, hemosiderin-laden macrophage accumulation (not shown). Interstitial fibrosis, granulomatous inflammation or capillaritis was not observed (Hematoxylin And Eosin stain, magnification ×100—a and ×200—b).

Treatment with high doses of oral prednisone (0.75 mg/kg of weight) as a monotherapy was adopted for 6 weeks and gradually tapered to 0.5 mg/kr for another 6 weeks and 20 mgr for another 6 weeks leading to a profound improvement of symptoms (dyspnoea) as well as imaging and laboratory findings including complete resolution of bilateral areas of ground-glass opacities (Figure 3(b)) as well as a significant incline of his haemoglobin levels and decrease of his CRP and ESR titers, respectively. The patient is now followed for almost 3 months, on an outpatient basis, in good clinical condition, free of disease relapses, afebrile, and hemodynamically stable on 10 mgr/day of oral corticosteroids. Due to high incidence of relapses, the patient is under close monitoring.

## 3. Discussion

This is one of the few IPH cases reported in literature affecting a previously healthy adult male patient, since the disease affects mostly children [6, 7]. Our patient presented with relapsing episodes of fever accompanied with general fatigue mainly attributed to the iron deficiency anemia. Despite the usual disease manifestation, hemoptysis was not present in this case. After thorough examination including extensive laboratory tests, gastrointestinal workup, chest HRCT scan, and BAL resulting in the exclusion of other causes of DAH, diagnosis of IPH was confirmed by VATS lung biopsy. Patient was then treated with high doses of oral corticosteroids and exhibited excellent clinical, laboratory, and imaging responsiveness. 

IPH is a very rare condition affecting mostly children and causing recurrent episodes of DAH that may lead to lung fibrosis [4, 6, 7]. Although its pathogenesis remains elusive and controversial, positive response to immunosuppressive therapeutic approaches suggests an immune system involvement [8]. This notion is supported by studies showing that one out of four children with IPH who survive develop immune disorders [9], while three out of four children present with circulating C1q-binding immune complexes [10]. Nevertheless, results from lung biopsy studies seem rather contradictory since they fail to reveal the accumulation of immune complexes or other findings compatible with immune derangement [11–13]. Intriguingly, IPH is often accompanied by celiac disease and has been reported that a gluten-free diet could be proven beneficial [14–17]. In addition, a possible connection between IPH and infectious agents has also been reported [18].

Clinical presentation of IPH is similar to that of any other cause of DAH. Anaemia is present, caused by the loss of blood to the pulmonary interstitium. Dyspnoea and cough are common in children, with failure to thrive observed in most cases [4, 19]. Adults usually develop dyspnoea and fatigue on exertion, resulting from the development of iron deficiency anaemia. Haemoptysis is a common finding in IPH patients irrespective of age, although it is more commonly presented in adult patients [5, 20]. 

During acute DAH, chest CT reveals diffuse lung infiltrates and pulmonary function tests (PFTs) are characterized by an increase in diffusion capacity for carbon monoxide, highly indicative of alveolar haemorrhage. BAL can be of major help to set the diagnosis of IPH in the absence of other causes of DAH since it may reveal the presence of numerous hemosiderin-laden macrophages (siderophages) suggestive of alveolar hemorrhage [1]. Due to lack of pathognomonic findings, IPH diagnosis is established upon exclusion of all other possible causes of DAH [4, 21], in combination with specific pathologic findings revealing bland alveolar haemorrhage with absence of vasculitis and/or accumulation of immune complexes within lung parenchyma [22, 23].

Regarding IPH treatment, a number of therapeutic approaches have been applied with conflicting results. Disease rareness and the absence of firm diagnostic criteria preclude the existence of randomized controlled trials estimating the efficacy of immunomodulatory and/or anti-inflammatory agents for IPH. Therefore, treatment strategy is based upon small case series or case reports [7, 8, 24, 25]. Corticosteroids still represent the cornerstone of IPH therapeutic strategy. Results from individual case studies, as well as case series studies, report remission of pulmonary bleeding as well as higher survival rates and slower pulmonary fibrosis progression following treatment with corticosteroids of different therapeutic regimens ranging from 0.5 mg/kg/day to 2 mg/kd/day during acute symptoms and tapering after remission [4, 8, 24–26]. Azathioprine and hydroxychloroquine have also been used in a small number of patients with steroid-refractory disease with favourable results [27–31]. On two cases, lung transplantation was performed but bleeding recurrence within the allograft discouraged future attempts [32, 33]. Despite the positive effect of immunosuppressive therapy for most patients, about 14–29% of them die from acute or to chronic respiratory failure [7, 26]. The course of IPH appears to be more severe in children [7]. Adults seem to have a more prolonged survival [26]. Patients usually have more than one acute episodes of DAH. Those who survive tend to develop pulmonary fibrosis due to recurrent intrapulmonary bleeding, associated with exertional dyspnoea and chronic anaemia.

In our case, the patient did not exhibit a typical clinical pattern of DAH. He was neither dyspnoeic, nor he reported any haemoptysis event. The clinical suspicion of a haemorrhage was raised by the continuously decreasing haemoglobin level and the absence of any other possible site of bleeding and his chest HRCT. BAL was revealing and confirmed the diagnosis of DAH. We, therefore, performed a thorough laboratory evaluation aiming at identifying the underlying cause of the bleeding. His immunologic profile was negative, leading us to the notion that IPH could be the causative condition. In line with this, a lung biopsy was performed and extensively reviewed by an expert pathologist, revealing a bland alveolar haemorrhage pattern with no specific pathology. Additionally, an immunologic study of the biopsy failed to show any immune complexes on the tissue sample. Subsequent therapeutic approach with high doses of corticosteroids was successful, leading to patient dismission. Currently, one year after initiation of corticosteroids, the patient is under no treatment and free of relapses. The above findings were enough to establish the diagnosis of IPH. Nevertheless, it is of utter importance to stress that DAH is a challenging, acute condition requiring early recognition of DAH along with identification of the underlying syndrome and appropriate treatment to achieve optimal results.
